# Pre-transplant CD69+ extracellular vesicles are negatively correlated with active ATLG serum levels and associate with the onset of GVHD in allogeneic HSCT patients

**DOI:** 10.3389/fimmu.2022.1058739

**Published:** 2023-01-13

**Authors:** Gianluca Storci, Francesco Barbato, Francesca Ricci, Pier Luigi Tazzari, Serena De Matteis, Enrica Tomassini, Michele Dicataldo, Noemi Laprovitera, Mario Arpinati, Margherita Ursi, Enrico Maffini, Elena Campanini, Elisa Dan, Silvia Manfroi, Spartaco Santi, Manuela Ferracin, Massimiliano Bonafe, Francesca Bonifazi

**Affiliations:** ^1^ IRCCS Azienda Ospedaliero-Universitaria di Bologna, Bologna, Italy; ^2^ Department of Experimental, Diagnostic and Specialty Medicine (DIMES) University of Bologna, Bologna, Italy; ^3^ Consiglio Nazionale delle Ricerche (CNR) Institute of Molecular Genetics "Luigi Luca Cavalli-Sforza", Bologna, Italy; ^4^ IRCCS, Istituto Ortopedico Rizzoli, Bologna, Italy

**Keywords:** anti-T lymphocyte globulin, graft versus host disease, extracellular vesicles, CD69, CD103

## Abstract

Graft versus host disease (GVHD) is a major complication of allogeneic hematopoietic stem cell transplantation (HSCT). Rabbit anti-T lymphocyte globulin (ATLG) in addition to calcineurin inhibitors and antimetabolites is a suitable strategy to prevent GVHD in several transplant settings. Randomized studies already demonstrated its efficacy in terms of GVHD prevention, although the effect on relapse remains the major concern for a wider use. Tailoring of ATLG dose on host characteristics is expected to minimize its side effects (immunological reconstitution, relapse, and infections). Here, day -6 to day +15 pharmacokinetics of active ATLG serum level was first assayed in an explorative cohort of 23 patients by testing the ability of the polyclonal serum to bind antigens on human leukocytes. Significantly lower levels of serum active ATLG were found in the patients who developed GVHD (ATLG_AUC_CD45_: 241.52 ± 152.16 vs. 766.63 +/- 283.52 (μg*day)/ml, p = 1.46e^-5^). Consistent results were obtained when the ATLG binding capacity was assessed on CD3+ and CD3+/CD4+ T lymphocytes (ATLG_AUC_CD3_: 335.83 ± 208.15 vs. 903.54 ± 378.78 (μg*day)/ml, p = 1.92e^-4^; ATLG_AUC_CD4_: 317.75 ± 170.70 vs. 910.54 ± 353.35 (μg*day)/ml, p = 3.78e^-5^. Concomitantly, at pre-infusion time points, increased concentrations of CD69+ extracellular vesicles (EVs) were found in patients who developed GVHD (mean fold 9.01 ± 1.33; p = 2.12e^-5^). Consistent results were obtained in a validation cohort of 12 additional ATLG-treated HSCT patients. Serum CD69+ EVs were mainly represented in the nano (i.e. 100 nm in diameter) EV compartment and expressed the leukocyte marker CD45, the EV markers CD9 and CD63, and CD103, a marker of tissue-resident memory T cells. The latter are expected to set up a host pro-inflammatory cell compartment that can survive in the recipient for years after conditioning regimen and contribute to GVHD pathogenesis. In summary, high levels of CD69+ EVs are significantly correlated with an increased risk of GVHD, and they may be proposed as a tool to tailor ATLG dose for personalized GVHD prevention.

## Introduction

Graft versus host disease (GVHD) is a major complication of allogeneic hematopoietic stem cell transplantation (HSCT). Although risk factors for GVHD have been already described, the prediction of GVHD occurrence at the single patient level is still far from clinical practice. Several attempts have been done in the recent years to decrease the incidence of GVHD, such as the use of rabbit anti-thymocyte globulin (ATG)/anti-T lympho-globulin (ATLG), post-transplant cyclophosphamide, m-TOR inhibitors, and new *ex vivo* T- cell depletion strategies (1, 2). The mechanisms of action of ATG/ATLG are still partially unknown: *in vivo* T- and B-cell depletion and inhibition of inflammatory cells and dendritic cells are regarded as putative mechanisms of drug action (3–6). ATG/ATLG polyclonal serum contains at least two IgG fractions: non-specific rabbit IgGs against non-human antigens and specific (active) IgGs against a wide array of human lymphocyte antigens (7–10). Active ATLG is expected to elicit the depletion and anergy on both host and infused T cells (3, 11, 12), and it can be measured by cytofluorimetric assays that detect the capability of the polyclonal serum to bind primary lymphocytes or lymphoblastoid cell lines (13–16). A pharmacokinetic approach has been proposed as an appropriate strategy to tailor the dose of polyclonal sera and to maximize the benefit/risk ratio of such GVHD prevention (17–20). The rationale of this approach relies on the tenet that the binding of cellular ATG/ATLG targets can modify the free active compartment of the drug and, ultimately, its overall clinical impact (21). In fact, heterogeneity in the drug kinetics can explain the inter-individual variability in drug response (15, 17, 18). Under this perspective, the pre-transplant host inflammatory status, which depends upon the activation and the burden of immune cells that survive the conditioning regimen, is likely to play a role in GVHD pathogenesis and to affect the efficacy of GVHD prophylaxis (22). Recent results show that host tissue resident memory T cells (TRM_T cells) are a pro-inflammatory compartment that is resistant to the conditioning regimen and exert a primary role in GVHD (23–25). TRM_T cells can be identified, among others, by the expression of the type II transmembrane receptor CD69, aka CLEC2C, a T-cell activation marker which belongs to the lectin superfamily, which acts as regulator of T cell homeostasis and tissue egress, and by CD103, aka ITGAE, an alpha integrin that binds cadherin, a protein typically expressed on epithelial cells (26–28). TRM_T cells are highly enriched in epithelial tissue compartments, i.e. the skin and the gut, the body districts in which GVHD mainly occurs (26–32). However, being tissue cells, TRM_T cells are very difficult to detect in the peripheral blood (28–32), even more during ATLG prophylaxis which induces profound lymphopenia (23). In principle, it can be assumed that if TRM_T specific markers can freely diffuse in the plasma/serum, such molecules may be taken as surrogate markers of the host TRM_T burden. Extracellular vesicles (EVs) are suitable candidates to play a primary role in the scenario described above, as they may originate from membrane budding and may carry molecules on their surface that can identify the cell of origin (33). Reasonably, TRM_T cells can be tracked by assessing the cognate EVs in body fluids (33). Here we addressed the issue of the relationship between the kinetics of serum CD69+ EVs and ATLG pharmacokinetics in the pre-transplant phase in order to investigate if CD69+ EVs represent a marker of GVHD as well as ATLG pharmacokinetics.

## Material and methods

### Patient characteristics

All patients suffering from hematological malignancies undergoing allogeneic HSCT at IRCCS AOU S. Orsola-Malpighi of Bologna were enrolled in a prospective monocenter observational study (MET_SCT_2018; 151/2018/Sper/AOUBO; NCT 03871269), aimed to find biological markers of transplant complications. Inclusion criteria were age (>17 years), any type of allogeneic HSCTs, and written informed consent. In the present analysis, patients transplanted from related and unrelated donors and receiving ATLG as GVHD prophylaxis were included. GVHD prevention was given with calcineurin inhibitor (cyclosporin or tacrolimus) and antimetabolite (either methotrexate or mycophenolate mophetil) and ATLG (Grafalon, Neovii, Switzerland) at a total dose of 30 mg/kg from days -6 to -2 in a 12-h infusion *via* a high-flow central catheter. Acute and chronic GVHD were graded according to the standard criteria (34, 35). Patients with AML and ALL in first remission were considered as early disease phases; all the remaining patients were named as being in advanced phase. Two cohorts of HSCT patients were studied in this investigation: Group 1 (23 patients) was regarded as the explorative cohort; Group 2 (12 patients) was regarded as the validation cohort.

### Serum samples sampling

In the explorative cohort, peripheral blood sample collection was scheduled at 11 time points, as follows: days -6, -5, -4, -3, -2 (before HSCT); day 0 (the day of HSCT); and days +3, +6, +9, +12, +15. In the validation cohort, peripheral blood sample collection was scheduled at three time points, as follows: days -6 and -3 (before HSCT) and day 0 (the day of HSCT). The samples were centrifuged at 1500×*g* for 15 minutes within 4 h of blood withdrawal. Sera were immediately anonymized and stored at -80°C.

### Active ATLG serum level quantification

**Figure 1:**
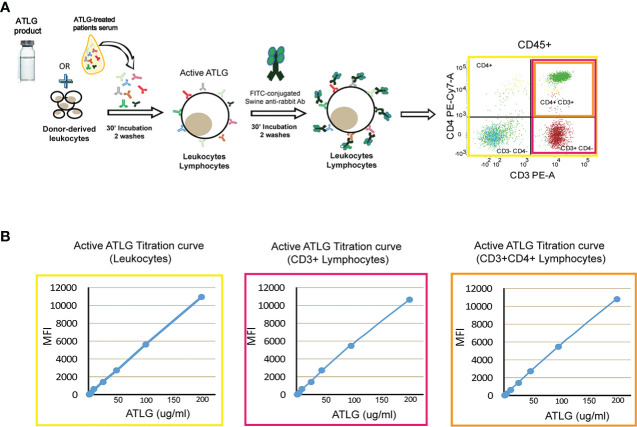
Active ATLG binding assay. **(A)** Schematic representation of flow cytometry analysis to measure active ATLG. Serum from ATLG-treated patients is incubated with healthy donor-derived leukocytes. Active ATLG is detected by FITC-conjugated swine anti-rabbit antibody on CD45+ cells, CD3+, and CD3+/CD4+ lymphocytes. **(B)** Calibration curve [mean fluorescent intensity vs. ATLG concentration, (μg/ml)] on CD45+ cells, CD3+ and CD3+/CD4+ lymphocytes.

A cytofluorimetric approach was set up in order to measure the amount of serum active ATLG concentration as follows: briefly, 10 ml of EDTA-anticoagulated whole blood was obtained by venipuncture from pools for at least four healthy blood donors at each time of assay. Samples were diluted 1:2 in phosphate-buffered saline (PBS), gradient-separated by Ficoll-Hypaque at 400×*g* for 25 min, and counted and resuspended in PBS at a final concentration of 5e^+5^ cells/ml. The calibration curve was obtained by serial dilutions of ATLG (200 μg/ml, 100 μg/ml, 50 μg/ml, 25 μg/ml, 12.5 μg/ml, 6.25 μg/ml, 3.125 μg/ml, 1.56 μg/ml) in PBS. Mononuclear cells were then incubated for 30 min at room temperature with 50 μl of each ATLG dilution or with 50 μl of patients’ serum. After washing twice with sterile PBS, 5 μl of FITC-conjugated swine-anti-rabbit IgG (Dakopatt, Copenhagen, Denmark) was added to each sample, together with anti CD45-PercP Cy5.5, CD3-PE, CD4-PE-Cy7, and mAbs (Becton Dickinson, San Jose, CA). After 30 min the samples were washed twice with PBS and finally resuspended in 500 μl of PBS (see **Figure 1A**). Samples were analyzed on a FACS Lyric equipment (Becton Dickinson, San Jose, CA). ATLG mean fluorescence intensity (MFI) was assessed on CD45+ cells and gated on CD3+ and CD4+ lymphocytes and therefore converted into μg/ml by the calibration curve (see **Figure 1B**). The exposure to ATLG was then expressed as area under the curve (AUC, 36)

### Jurkat T cell line *in vitro* culture

The Jurkat T cell line was cultured in RPMI (Euroclone, Italy) supplemented with 10% FBS, penicillin/streptomycin 1% and L-Glutamine 1%. Phytoaemagglutinin (PHA) was added at 1μg/ml and 5μg/ml for 72h. The culture supernatant was harvested, centrifuged at 2500xg for 15 minutes to clear cell debris, aliquoted and stored at -80°C until the day of analysis.

### Extracellular vesicle FACS analysis

EVs were assessed and quantified by cytofluorimetric assays performed on different platforms, i.e. Lyric (Becton Dickinson, San Jose, CA, and Cytoflex (Beckman Coulter, Brea, CA). On the former platform the EV protocol applied refers to that described previously (33). Briefly, 5 μl of patients’ serum were added to 95 μl of filtered PBS and 95 μl of a mix of fluorochrome conjugated moAbs, namely CD45-BV510 and CD69-APC H7 (Becton Dickinson, San Jose, CA). FITC-conjugated phalloidin and APC-conjugated Lipophilic Cationic Dye (Becton Dickinson, San Jose, CA) were used to exclude non-EV events from the analysis. The number of CD69+ EV/ml was assessed using the TruCount BD system (Becton Dickinson, San Jose, CA). The dimensional and phenotypic analysis of the CD69+/CD103+ EVs (CD69 APC-H7 clone FN50 and CD103 BV510 clone BerAC8; Becton Dickinson, San Jose, USA) and CD9+ and CD63+ (CD9 PE and CD63-BV510 clone H5C6) Becton Dickinson, USA) EVs were analyzed through flow cytometric analysis and carried out using the Megamix-PLUS FSC Kit (BioCytex, Marseille, France) following the manufacturer instructions.

### Stochastic optical reconstruction microscopy

Single-molecule super-resolution microscopy for CD69+EVs serum-derived exosomes was performed as previously described (37). Briefly, 1 ml of serum was serially centrifuged for 10 min at 300×*g* and for 20 min at 1200×*g* in order to eliminate cell debris. EVs were isolated by *ad hoc* anti-CD9 conjugated latex beads (Hansa_Biomed, Tallin, Estonia) according to manufacturer instructions. EVs were fixed in 3% PFA and stained in suspension with mouse anti-CD69 1:200 (1 h at RT), followed by AlexaFluor-647 Goat anti-Mouse IgGs (Molecular Probes, Thermo Fisher) 1:100 (1 h at RT). After each step the samples were centrifuged at 6,000×*g* for 10 min at RT. For acquisition, 5 μl of the labeled EV pool was deposited in a petri dish (CellView) and air-dried under the laminar hood for 30 min before adding 250 μl of freshly prepared STORM buffer (Abbelight, France). Acquisitions were performed on an N-STORM instrument (Nikon Instruments, Milan, Italy) with a DU-897 EM-CCD camera (Andor Technology) with ×100 TIRF (NA 1.49) objective, coupled with 10-mW 647 excitation/reported laser (CrystaLaser) used at 80% power for 10,000 frames/acquisition. Data reconstruction was obtained with the STORM analysis module of the NIS-Elements software v.5.31.

### Transmission electron microscopy

For transmission electron microscopy analysis, 1 ml of serum was serially centrifuged 10 minutes at 300×*g* and 20 min at 1200×*g* in order to eliminate cell debris. EVs were isolated by *ad hoc* anti-CD9 conjugated latex beads (Hansa_Biomed, Tallin, Estonia) according to manufacturer instructions. EVs were fixed with 3% PFA in phosphate buffer and stained in suspension with mouse anti-CD69 (1:200) followed by 5-nm gold-anti-mouse (BBI International, 1:200); 5 µl of suspension was deposited on 200 mesh Formvar-carbon-coated grids and left to absorb for 20 min at room temperature. The observations were carried out with a JEOLJEM-1011 (Jeol Jem, Peabody, MA, USA) transmission electron microscope operated at 100 kV.

### Luminex assay for class 1 HLA antigen typization

We added 5 μl of Immuncor Class_I beads (Labscreen™ Single Antigen HLA Class I, One Lambda, CA, USA) to 20 μl of serum ATLG-treated HSCT patients in a 96-well plate (Corning Costar, NY, USA). After two washes with a specific buffer (Lab Screen Wash Buffer 10X), 100 μl of swine anti-rabbit IgG PE diluted 1:100 was added for 30 min. After three washes with specific buffer, samples were analyzed with system Ponent (Luminex, TX, USA) and analyzed using the Fusion Software (One Lambda, CA, USA).

### Capillary electrophoresis protein analysis

Capillary electrophoresis protein analysis of serum CD69+EV was performed in the protein simple equipment (Biotechne, MN, USA). Briefly, 1 ml of HSCT patient-derived serum was serially centrifuged, as follows: 1000×*g* for 15’, 2500×*g* for 15’, 20.000×*g* for 2 h, and 100.000×*g* for 3 h). The pellet was immediately extracted with RIPA buffer added with protease and phosphatase inhibitors (Roche); 500 ng of protein extract was run and analyzed for CD69 and CD63 protein content with specific antibodies (Clone FN50, Milteniy Biotech, Germany; and clone TS63, Thermo Fisher, respectively).

### Statistical analysis

Continuous variables were analyzed by *t*-test and Pearson correlation analysis. Repeated measures were analyzed using generalized linear mode (GLM) for repeated measures. Analysis of censored data was performed according to the Cox time-dependent model. Dichotomous variables were summarized as number and percentage and compared using the chi-square. All analyses were performed using the SPSS software package version 10 (SPSS Inc., Chicago, IL).

## Results

### Clinical outcome

Twenty-three consecutive patients were enrolled from September 2020 to February 2022 for the analysis as the explorative cohort. In this cohort median follow-up was 463 days (r: 48–645). Overall survival at 1 year was 82.6% (95% CI 60.1%–93.1%) while non-relapse mortality at 1 year was 13.0% (95% CI 3.3%–29.7%); cumulative incidence of aGVHD was 47.8% (95% CI 26.8%–66.1%) while cGVHD cumulative incidence was 17. 4% (95% CI 5.4%–35.0%). We then analyzed 12 patients enrolled from to July 2021 to January 2022 as the validation cohort. Median follow-up in this cohort was 272 days (r: 200–399). All patients were still alive at last follow-up. Cumulative incidence of aGVHD was 41.7% (95% CI 19.9%–73.0%) while cGVHD cumulative incidence was 17.5% (95% CI 4.7%–53.9%). The whole analyzed population included 35 patients and median follow-up was 421 (r: 48–645). Overall survival and non-relapse mortality at 1 year in the whole population were, respectively, 87.1% (95% CI 68.5%–95.1%) and 8.6% (95% CI 2.1%–20.8%). Overall incidence of aGVHD and cGVHD were, respectively, 45.9% (95% CI 28.6%–61.5%) and 18.7% (95% CI 3.8%–27.6%). The clinical and transplant characteristics of the explorative and validation cohorts and of the whole population are reported in **Table 1**. Factors univariately tested for correlation with aGVHD were patient’s age, disease phase, sex mismatch, Sorror score, BMI, HLA mismatches, and ALC pre ATLG infusion. The only variable associated with aGVHD was the HLA mismatch (**Table 2**).

**Table 1:** Clinical and transplant characteristics.

	Training cohort	Validation cohort	Total
**N°**	23	12	35
**Median age**	55 years (r: 18-69)	50 years (r: 28-69)	55 years (r: 18-69)
**Female (F)/Male(M)**	8/15	6/6	14/21
**Donor/Recipient sex mismatch (F/M)**	n=2 (8.7%)	n=1 (8.3%)	n=3 (8.6%)
Diagnosis			
AML ALL MDS LYMHOMAS OTHERS	n=9 (39.1%) n=4 (17.4%) n=4 (17.4%) n=2 (8.7%) n=4 (17.4%)	n=5 (41.7%) n=2 (16.7%) n=1 (8.3%) n=2 (16.7%) n=2 (16.7%)	n=14 (40.0%) n=6 (17.1%) n=5 (14.2%) n=4 (11.4%) n=6 (17.1%)
Phase at transplant			
Early Advanced	n=13 (56.5%) n=10 (43.5%)	n=4 (33.4%) n=8 (66.6%)	n=17 (48.6%) n=18 (51.4%)
HLA matching			
HLA 10/10 HLA 9/10 HLA 8/10 HLA 8/8 HLA 7/8	n=14 (60.8%) n=8 (34.8%) n=1 (4.4%) n=16 (69.6%) n=7 (30.4%)	n=7 (58.3%) n=3 (25.0%) n=2 (16.7%) n=7 (58.3%) n=5 (41.7%)	n=21 (60.0%) n=11 (31.4%) n=3 (8.6%) n=23 (65.7%) n=12 (34.3%)
Donor type			
Matched Related Matched Unrelated* Mismatched Unelated	n=1 (4.4%) n=13(56.5%) n=9 (39.1%)	n=1 (8.3%) n=6 (50.0%) n=5 (41.7%)	n=2 (5.7%) n=19 (54.3%) n=14 (40.0%)
Stem Cell Source			
PBSC	n=23 (100%)	n=12 (100%)	n=35 (100%)
Conditioning intensity:			
myeloablative reduced intensity	n=12 (52.2%) n=11 (47.8%)	n=7 (58.3%) n=5 (41.7%)	n=19 (54.3) n=16 (45.7%)
GVHD Prophylaxis			
CSA-MTX-rATLG CSA-MMF-rATLG	n=21 (91.3%) n=2 (8.7%)	n=12 (100%) n=0	n=33 (94.3%) n=2 (5.7%)
**Acute GVHD**	n=11 (47.8%)	n=6 (50.0%)	n=17 (48.7%)
Grade 1 Grade 2 Grade 3 Grade 4 Median time of onset (range)	n=3 (13.0%) n=3 (13.0%) n=3 (13.0%) n=2 (8.8%) 36 days (r: 11-157)	n=2 (16.7%) n=3 (25.0%) n=1 (8.3%) n=0 34 days (r: 17-129)	n=5 (14.3%) n=6 (17.1%) n=4 (11.4%) n=2 (5.7%) 36 (r: 11-157)
**Chronic GVHD**	n=4 (17.4%)	n=2 (16.7%)	n=6 (17.1%)
Mild Moderate Severe Median time of onset (range)	n=2 (8.7%) n=1 (4.9%) n=1 (4.9%) 119 days (r: 53-328)	n=0 (0.0%) n=0 (0.0%) n=2 (16.7%) 183 days (r: 139-227)	n=2 (5.7%) n=1 (2.8%) n=3 (8.6%) 158 (r: 56-328)
Acute/Chronic GVHD combination			
Acute or Chronic GvHD Acute and Chronic GvHD Only Acute GvHD Only Chronic GvHD	n=12 n=3 (25.0%) n=8 (66.7%) n=1 (8.3%)	n=6 (50.0%) n=2 (16.7%) n=4 (33.3%) n=0	n=18 (51.4%) n=5 (14.3%) n=12 (34.3%) n=1 (2.8%)
**Median Follow Up time(range)**	463 days (r: 48-645)	272 days (r: 200-399)	421 (r: 48-645)

AML, acute myeloid leukemia; ALL, acute lymphoblastic leukemia; MDS, myelodysplastic syndrome; HLA, human leukocyte antigen; GVHD, graft versus host disease; CSA, Ciclosporin; MTX, methotrexate; rATLG, rabbit anti-T –lymphoglobulin.

**Table 2:** Multivariate analysis.

Factor	HR	CI 95%	p VALUE
**Donor age**	0.970	0.901-1.045	0.424
**Recipient age**	1.007	0.952-1.065	0.816
**Sex mismatch**	2.462	0.316-19.147	0.389
**HLA mismatch**	0.309	0.097-0.986	**0.047**
**Disease phase**	0.904	0.290-2.816	0.862
**HCT-CI**	1.212	0.390-3.764	0.739
**ALC pre-rATLG**	1.236	0.889-1.717	0.207

HLA, Human Leukocyte Antigen; HCT-CI, Hematopoietic Cell Transplantation-specific Comorbidity Index; ALC, Absolute Lymphocyte Count; rATLG, rabbit anti-T –lymphoglobulin. Bold means statistically significant.

### Reduced pre-transplant active ATLG serum level is predictive of GVHD

**Figure 2:**
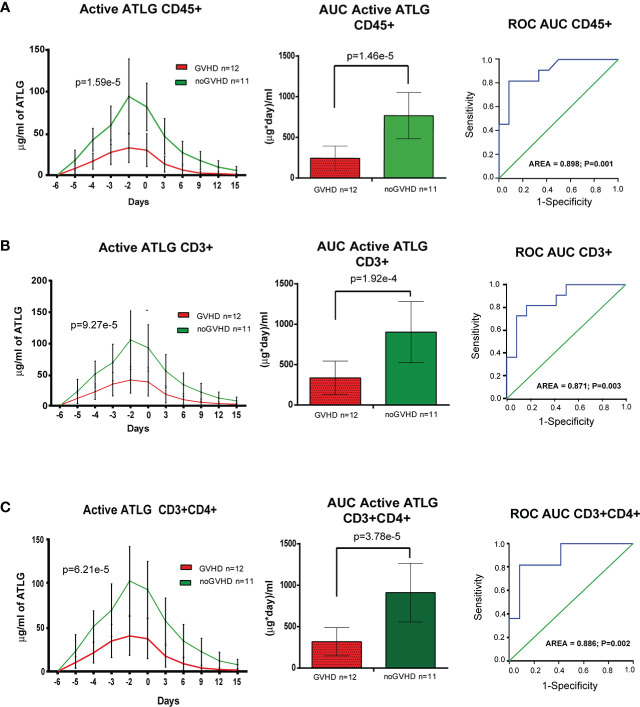
Pharmacokinetic analysis of active ATLG. **(A)**, Pharmacokinetic analysis (left panel), AUC (middle panel) and ROC analysis (right panel) of active ATLG bound to CD45+cells from day -6 to day +15. **(B)**, Pharmacokinetic analysis (left panel), AUC (middle panel) and ROC analysis (right panel) of active ATLG bound to CD3+ lymphocytes from day -6 to day +15. **(C)**, Pharmacokinetic analysis (left panel), AUC (middle panel) and ROC analysis (right panel) of active ATLG bound CD3+/CD4+ lymphocytes from day -6 to day +15. The data are expressed as mean +/- S.D. p values refer to GLM for repeated measures, Left panels, and *t*-test for right panels.

The cytofluorimetric assay developed to quantify active ATLG serum level is depicted in **Figure 1**. The analysis conveyed a reduced active ATLG serum level in patients who developed any GVHD (GLM for repeated measures F = 30.995, p = 1.59e^-5^, **Figure 2A**, left panel). Consistently, reduced ATLG_AUC_CD45_ was found in GVHD patients (ATLG_AUC_CD45_: 241.52 +/- 152.16 vs. 766.63 +/- 283.52 (μg*day)/ml, *t*-test, p = 1.46e^-5^, **Figure 2A**, middle panel). Multivariate Cox analysis, including HLA mismatch (see **Table 2**) showed that ATLG_AUC_CD45_ is independently associated with GVHD (HR = 0.994, 95%CI, 0.990–0.998, p = 0.002). An ATLG_AUC_CD45_ below the median level (490.00 (μg*day)/ml) yields a substantially high risk of developing GVHD (HR = 19.509, 95% CI, 2.373–160.356, p = 0.006). Moreover, receiving operating characteristic (ROC) analysis showed that an ATLG_AUC_CD45_ = 349.50 μg/ml*day discriminated patients with or without GVHD with sensibility = 81.8% and sensitivity = 91.9% (**Figure 2A**, right panel). Notably, the cytofluorimetric assay was proven to be robust across different lymphocytes populations to show reduced active ATLG serum level in patients who developed GVHD: CD3+ T cells (GLM repeated measures F = 23.191, p = 9.27e^-5^ (**Figure 2B**, left panel); ATLG_AUC_CD3_: 335.83 ± 208.15 vs. 903.54 ± 378.78 (μg*day)/ml, *t*-test, p = 1.92e^-4^, **Figure 2B**, middle panel); ROC analysis showed that an ATLG_AUC_CD3 =_ 475.50 μg/ml*day discriminated patients with or without GVHD with sensibility = 81.8% and sensitivity = 83.3%, (**Figure 2B**, right panel); CD3+/CD4+ T cells (GLM repeated measures F = 24.848, p = 6.21e^-5^, **Figure 2C**, left panel; ATLG_AUC_CD4_: 317.75 ± 170.70 vs. 910.54 ± 353.35 (μg*day)/ml, *t*-test, p = 3.78e^-5^, **Figure 2C**, middle panel), ROC analysis showed that an ATLG_AUC_CD4_ = 394.5 μg/ml*day discriminated patients with or without GVHD with sensibility = 81.8% and sensitivity = 91.7%, (**Figure 2C**, right panel). Active ATLG serum levels were negatively correlated with the absolute lymphocyte count (ALC) at pre-ATLG infusion time point (day-6: ATLG_AUC_CD45_, r = -0.513, p = 0.015; ATLG-AUC_CD3_, r = -0.499, p = 0.018; ATLG-AUC_CD4,_ r = -0.492, p = 0.020), but not with BMI age, phase of disease, sex mismatch, HLA mismatch, and Sorror score (data not shown).

### Serum CD69+EVs as potential tool to measure the recipient TRM_T burden

**Figure 3:**
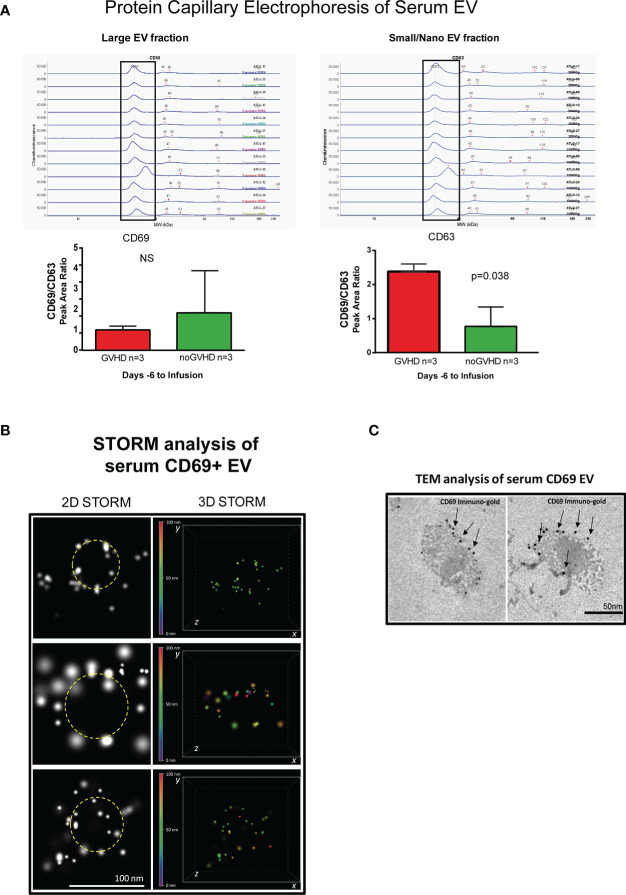
Detection of serum CD69+ EVs. **(A)**, Capillary electrophoresis protein analysis of EVs separated by ultracentrifugation at 100.000×*g* (small/nano EVs fraction) and 20.000×*g* (Large EVs fraction). **(B)** STORM analysis of serum EVs isolated by anti-CD9-conjugated latex beads pull-down. **(C)**, Immunogold-specific CD69 electron microscopy analysis of EVs isolated by anti-CD9-conjugated latex beads pull-down. ns, not significant.

**Figure 4:**
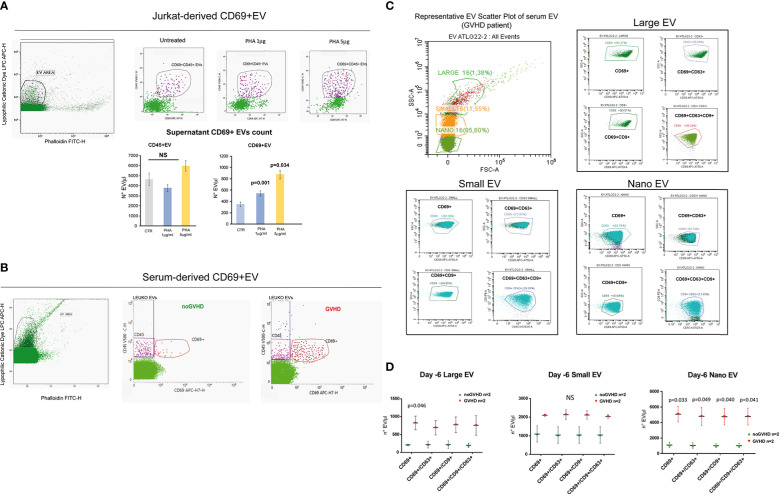
Phenotypic characteristics of CD69+ EVs in HSCT samples. **(A)** Representative gating strategy for EV detection from un-stimulated CD69+ Jurkat T cells and upon stimulation with PHA (1 or 5 μg/ml) for 72 h. Bar graphs represent the concentration of CD69+ EVs and CD45+ EVs in the culture supernatants. **(B)** Representative gating strategy for EV detection in the serum of HSCT patients. **(C)** Cytoflex analysis of serum EV in a representative GVHD patient. Plots refer to the concentration of CD69+, CD69+CD63+, CD69+CD9+, CD63+CD9+CD63+ among large (900 µm), small (160–500 nm), and nano (100 nm) EV fraction (see **Supplementary Figure 1**). **(D)**, Graphs charts of serum CD69+, CD69+CD63+, CD69+CD9+, CD63+CD9+CD63+ EVs according to size distribution in GVHD (n = 2) and noGVHD patients (n = 2). Values are expressed as number of EV/μl. Data are represented as mean +/- S.D. NS, Not Significant.

**Figure 5:**
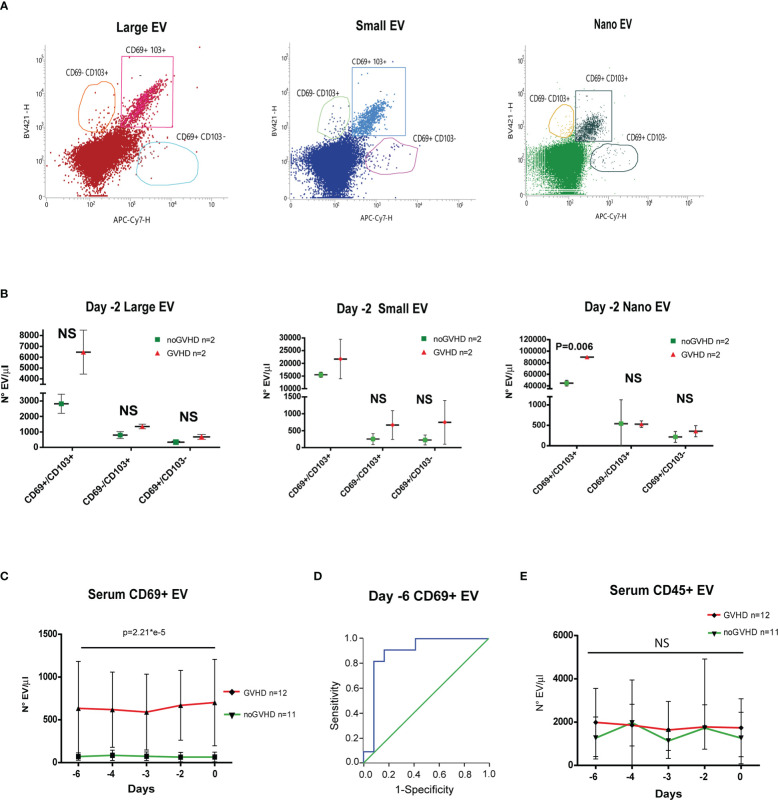
Evaluation of CD69+ EVs in HSCT samples. **(A)** Representative FACS analysis of serum EV size distribution and CD69/CD103 expression, in GVHD and noGVHD patients at -2 day. **(B)**, Dot Plot of serum CD69+/CD103-, CD69+/CD103+, CD69-/CD103+ EVs according to size distribution in GVHD (n = 2) and noGVHD (n = 2) patients at day-2. **(C)** FACS analysis of day -6 to day 0 serum CD69+ EVs in GVHD (n = 12) and noGVHD (n = 11) patients. **(D)**, ROC analysis of serum CD69+ EVs in GVHD and noGVHD patients. **(E)** FACS analysis of day -6 to day 0 serum CD45+ EVs in GVHD (n = 12) and noGVHD (n = 11) patients. Values are expressed as number of EVs/μl. Data are reported as mean +/- S.D.; p refers to GLM analysis for repeated measures. NS, Not Significant.

Recent papers showed that recipient CD69+TRM_T cells are predictive of GVHD in HSCT patients (24). We here investigated whether CD69+EVs may be conceived as surrogate markers of host CD69+ TRM_T cells burden in the pre-transplant phase and thus regarded as predictive markers of GVHD in ATLG-treated patients. Purposely, 100.000×*g* nano/small EVs and 20.000×*g* large EV fractions were separated by ultracentrifugation from three GVHD and three noGVHD serum samples. By means of capillary electrophoresis protein analysis, we show higher CD69 protein level (expressed as ratio over the EV marker CD63) in the small/nano EV fraction of GVHD patients’ serum, compared to the same EV compartment in noGVHD serum samples (**Figure 3A**). Notably, the difference is not detectable in the large EV serum fraction (see **Figure 3A**). We then obtained representative pictures of CD69+ EVs from a GVHD patient’s serum EVs captured by anti-CD9-conjugated latex beads, assessed by STORM and TEM analysis: the pictures show a diameter range of about 100 nm for CD69+ EVs, confirming that they belong to the nano EV fraction (**Figures 3B, C**). Thereafter, we set up cytofluorimetric assays to specifically detect CD69+ EVs (33). We first aimed at verifying that CD69+ T cells are capable to release CD69+ EVs. Purposely Jurkat T cells were cultured in the presence/absence of PHA for 72 h, a stimulus that triggers CD69 expression on the cell plasma membrane. The assay revealed a CD69+ EV population in the Jurkat cell culture supernatant, particularly in the PHA stimulated ones and that CD69+ EVs are predominantly CD45+, revealing their leukocytes origin (**Figure 4A**). The same protocol allowed us to show higher amounts of CD69+ EVs in GVHD patients’ serum samples (**Figure 4B**). Then, by means of the Cytoflex platform that easily discriminates nano (100 nm), small (160–500 nm), and large (900nm) EV fractions (**Supplementary Figure 1**), we found that CD69+ EVs co-express the EV markers CD9 and CD63, and that CD69+ EVs are more represented in GVHD patients serum samples, particularly in the nano EV compartment (**Figures 4C, D**). We then tested the hypothesis that CD69+ EVs also express the TRM-T marker CD103 (26–32). FACS analysis revealed that CD69+ EVs also carry the CD103 protein, and that the double-positive CD69+ CD103+ subset is the most represented EV population across the three EV compartments (**Figure 5A**). Consistent with the data above, the increased number of double-positive CD103+ CD69+ EVs in GVHD patients’ samples was predominately observed in the nano EV compartment (**Figure 5B**). Overall, we regarded these data as sufficient information to consider the serum level of CD69+ EVs as potential surrogate markers of the host CD69+ TRM_T burden. Prompted by the results above, we evaluated CD69+ EVs in the serum of the ATLG-treated HSCT patients (see **Table 1**). At all time points CD69+ EVs were significantly higher in patients who developed GVHD (mean fold 9.01 +/- 1.33; p = 2.1e^-5^, GLM for repeated measure, F = 11.381, p = 0.003, **Figure 5C**). ROC analysis showed that a concentration of CD69+ EVs = 238.5 particles/μl allows to discriminate patients who will developed GVHD with sensibility = 81.8% and sensitivity = 91.9%. (**Figure 5D**). Notably, the significant negative association between the concentration of CD69+ EVs at pre-ATLG administration time point (day -6) and active ATLG serum levels (ATLG_AUC_CD45,_ r = -0.469, p = 0.024; ATLG_AUC_CD3_, r = -0.436, p = 0.038; ATLG_AUC_CD4_, r = -0.455, p = 0.029, respectively) conveys that high CD69+ EV levels are a recipient’s feature that pre-exists ATLG prophylaxis. Interestingly, no difference was found when total CD45+ EVs were compared between the two patients’ groups (**Figure 5E**).

### Validation of ATLG kinetics and CD69+ EVs as markers of GVHD onset

**Figure 6:**
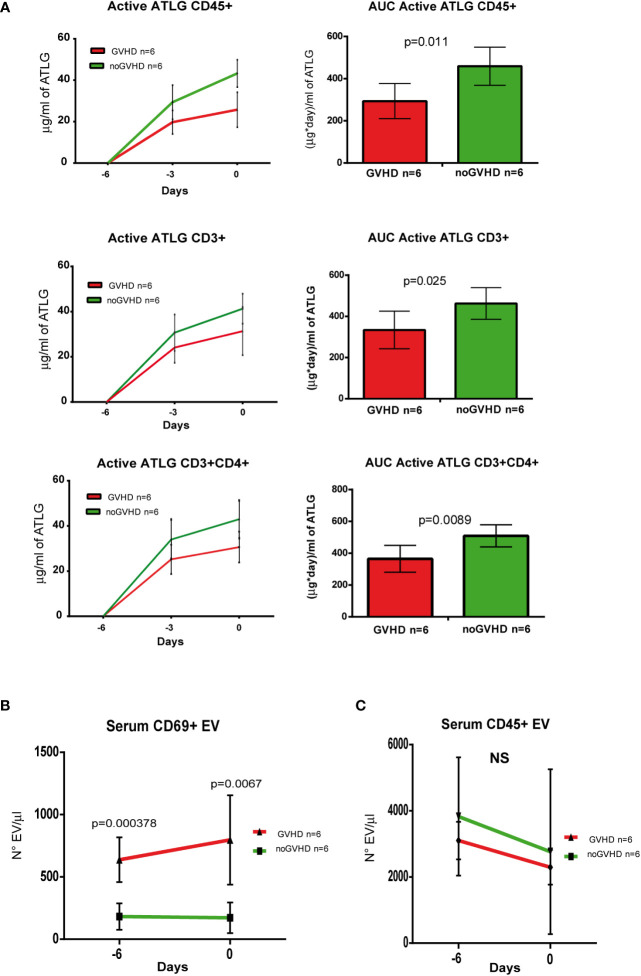
Validation of ATLG kinetics and CD69+ EVs as markers of GVHD onset. **(A)**, Pharmacokinetic analysis (left panel) and AUC (right panel) at day -6, day -3, and day 0 of active ATLG bound to: CD45+cells, CD3+ and CD3+/CD4+ lymphocytes; **(B)** FACS analysis of day -6 and day 0 serum CD69+ EVs in GVHD (n = 6) and noGVHD (n = 6) patients. **(C)** FACS analysis of day -6 and day 0 serum CD45+ EVs in GVHD (n = 6) and noGVHD (n = 6) patients. NS, Not Significant.

To validate the observations above, we assessed a validation cohort of 12 ATLG-treated HSCT patients. In the latter, the pre-transplant kinetics of ATLG (measured by three pre-transplant time points i.e. days -6, -3, and 0) showed the same pattern as in the explorative cohort, i.e., the serum level of active ATLG was lower in those patients who later developed GVHD (**Figure 6A**). Consistently, the concentration of pre-transplant serum CD69+ EVs, but not CD45+ EVs was higher in patients who developed GVHD, compared to noGVHD patients (**Figures 6B, C**). ROC analysis showed that CD69+ EV concentration of 384.50 EV/μl was able discriminate patients who developed GVHD with a sensibility of 100% and a sensitivity of 100%. These data confirm the capability of CD69+EVs in the pre-transplant phase to predict the GVHD onset in HSCT patients, even when measured in the pre-transplant phase.

## Discussion

Here we show that pre-transplant active ATLG serum levels follow different kinetics depending on whether patients will develop GVHD or not, i.e. lower active ATLG serum level is found in GVHD patients. As the administered dose of ATLG is the same among individuals, being based on body weight, the reduced serum levels of active ATLG in GVHD patients is likely to be the telltale of a higher amount of active ATLG sequestered by tissue antigens. A clear-cut demonstration of the phenomenon can be obtained by comparing the levels of Rabbit IgGs against the anti-HLA-A3 allele in HLA-A3 positive and negative patients after ATLG administration. Indeed, since that ATLG is obtained by rabbit immunization with the HLA-A3 homozygous Jurkat T cell line (**Supplementary Figure 2**), it is easy to demonstrate the presence of high levels of rabbit anti-HLA-A3 IgGs in the ATLG product and in the serum of HLA-A3 negative patients, as well as the almost complete absence of the rabbit IgGs in HLA-A3 positive ones (See **Supplementary Figure 2**) (38). These findings indicate that specific rabbit IgGs have been withdrawn from the active ATLG serum pool due to the binding to HLA in the tissues. Consequently, the presence of a tissue antigen for which ATLG possesses an affinity is able to almost completely deplete the cognate IgGs from the active ATLG serum pool. As previously reported, in this work we found a negative correlation between active ATLG serum levels and pre-infusion circulating lymphocyte count (21). On the basis of this relationship, ATLG administration has been proposed to be corrected by the number of circulating lymphocyte counts (18, 20). Circulating lymphocytes are a small subset (about 2 out of 100) of the overall lymphocyte burden in the body (39, 40). Circulating lymphocytes are likely to be in balance with their tissue counterparts; thus, it can speculated that recipients who show high levels of circulating lymphocytes might also be endowed with high burden of the cognate tissue-resident populations. By definition, tissue resident lymphocytes are very difficult to be assessed in the peripheral blood, especially after lymphodepletion when patients show profound lymphopenia. In this regard, it has been shown that TRM-T is resistant to conditioning regimens and persists in the recipients’ tissues for a long time, being able to set off GVHD (23–25). In particular, recent investigations revealed that recipient CD69+ TRM_T cells can promote GVHD upon the interaction with donor immune cells (23). Notably, we observed that ATLG contains IgGs that are able to bind CD69 antigen on T-cell surface (**Supplementary Figure 3**). It can be therefore hypothesized that lymphocytes are harbored in different tissues (such as the skin and the gut) and can be targeted by ATLG and that this phenomenon may differ among patients, thus impinging upon the chance of these cells to promote GVHD. In regard to this issue, we recently observed that CD69+ EVs take part to a complex EVs signature that characterizes GVHD patients even in the post-transplant phase (Burrello et al., manuscript in preparation). The major limitations of this study are the reduced number of patients, the monocentric design, and the heterogeneity of the diseases and conditioning. However, these latter variables are not predictive of GVHD occurrence, while GVHD prophylaxis was indeed homogeneous. Here, we show in two separate cohorts of HSCT patients that the pre-transplant kinetic of serum CD69+ EVs is predictive of GVHD. By means of different techniques, we demonstrated that serum CD69+EVs co-express classical EV markers (i.e. CD9 and CD63), are particularly enriched in the nano EVs compartment (around 100nm), and are of leukocyte origin (i.e. expressed the pan-leukocytes CD45+). Importantly, we also show that CD69+ EVs are CD103+, which represent a clear origin from TRM-T cells (31, 41). The striking negative correlation between CD69+EVs and free active ATLG serum level reinforces the notion that those patients who later developed GVHD are likely to be endowed with a burden of TRM-T cells, among which CD69+ cells may constitute a substantial compartment, and a potential target for ATLG. Hence, the measurement of serum CD69+ EVs can be considered a potential tool to tailor GVHD prophylaxis in ATLG-treated patients. A validation of our data in a larger case set and in a prospective larger study is warranted to confirm this hypothesis and to properly assess the sensibility and the sensitivity of CD69+ as predictors of GVHD.

## Data availability statement

The raw data supporting the conclusions of this article will be made available by the authors, without undue reservation.

## Ethics statement

The studies involving human participants were reviewed and approved by Comitato Etico Area Vasta Emilia Romagna, Codice protocollo: MET SCT 2018. The patients/participants provided their written informed consent to participate in this study.

## Author contributions

GS: Conception, design, writing, review data analysis, and interpretation; FBar: patient management and clinical Dataset management; FR: data acquisition; PT: data acquisition and interpretation; SDM: Manuscript revision and data acquisition; ET: data management and serum sampling programming; MD: patient management and clinical Dataset management; NL: data acquisition and manuscript revision; MA: patient management; MU: Patient management; EM: patient management; EC: HSCT handling and biobanking; ED: HSCT handling and biobanking; SM: Data acquisition; MF: data acquisition and manuscript revision; MB and FB: Conception, design, writing, review data analysis and interpretation, patients management. SS performs STORM and TEM analysis. All authors contributed to the article and approved the submitted version.
