# Supplementary figures and images for: Pre-transplant CD69+ extracellular vesicles are negatively correlated with active ATLG serum levels and associate with the onset of GVHD in allogeneic HSCT patients
Source: Front Immunol. 2023 Jan 13;13:1058739. doi: 10.3389/fimmu.2022.1058739 (PMC9880409; doi:10.3389/fimmu.2022.1058739)

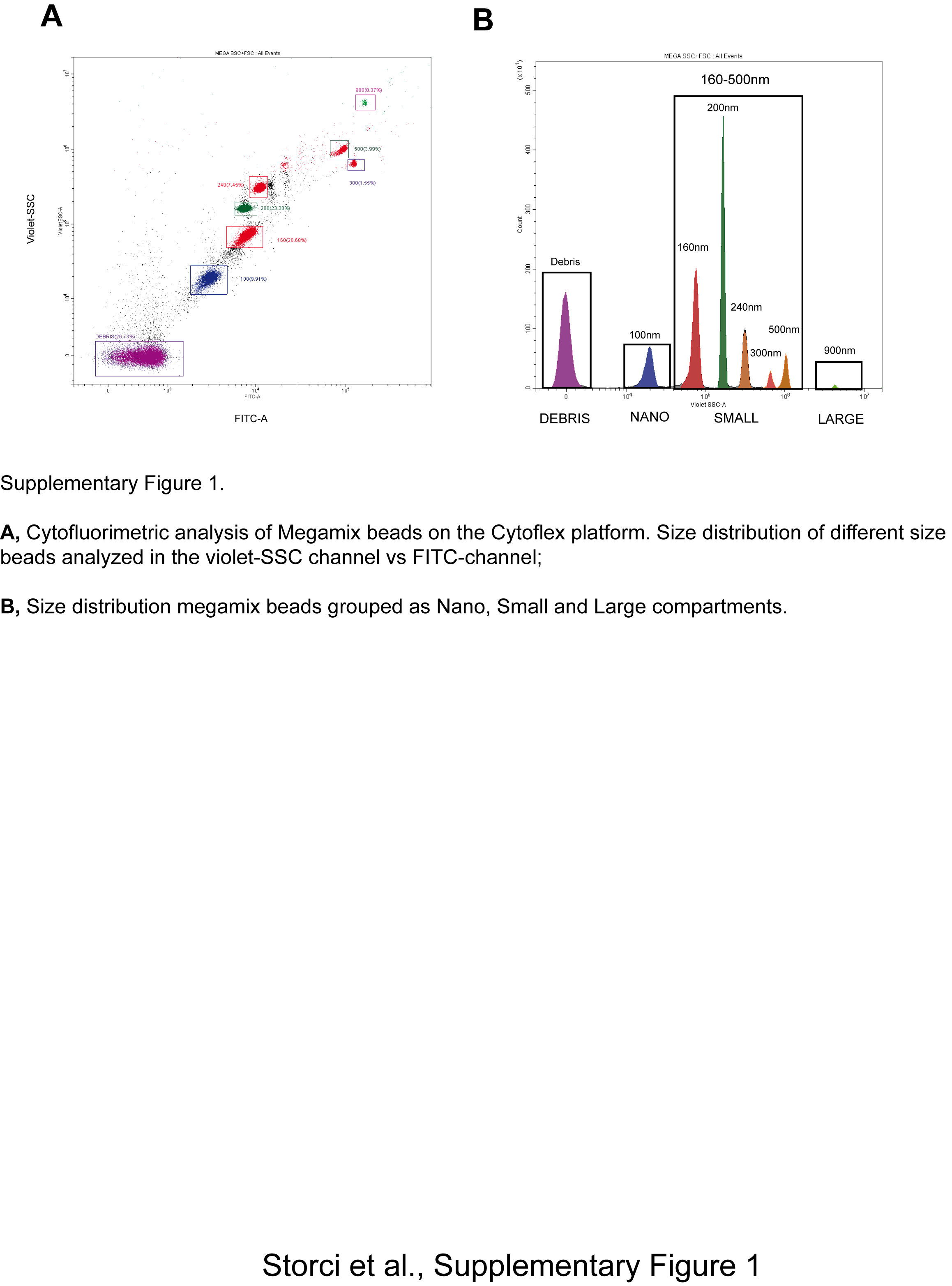

Supplement: Supplementary file 1 [file Image_1.tif]

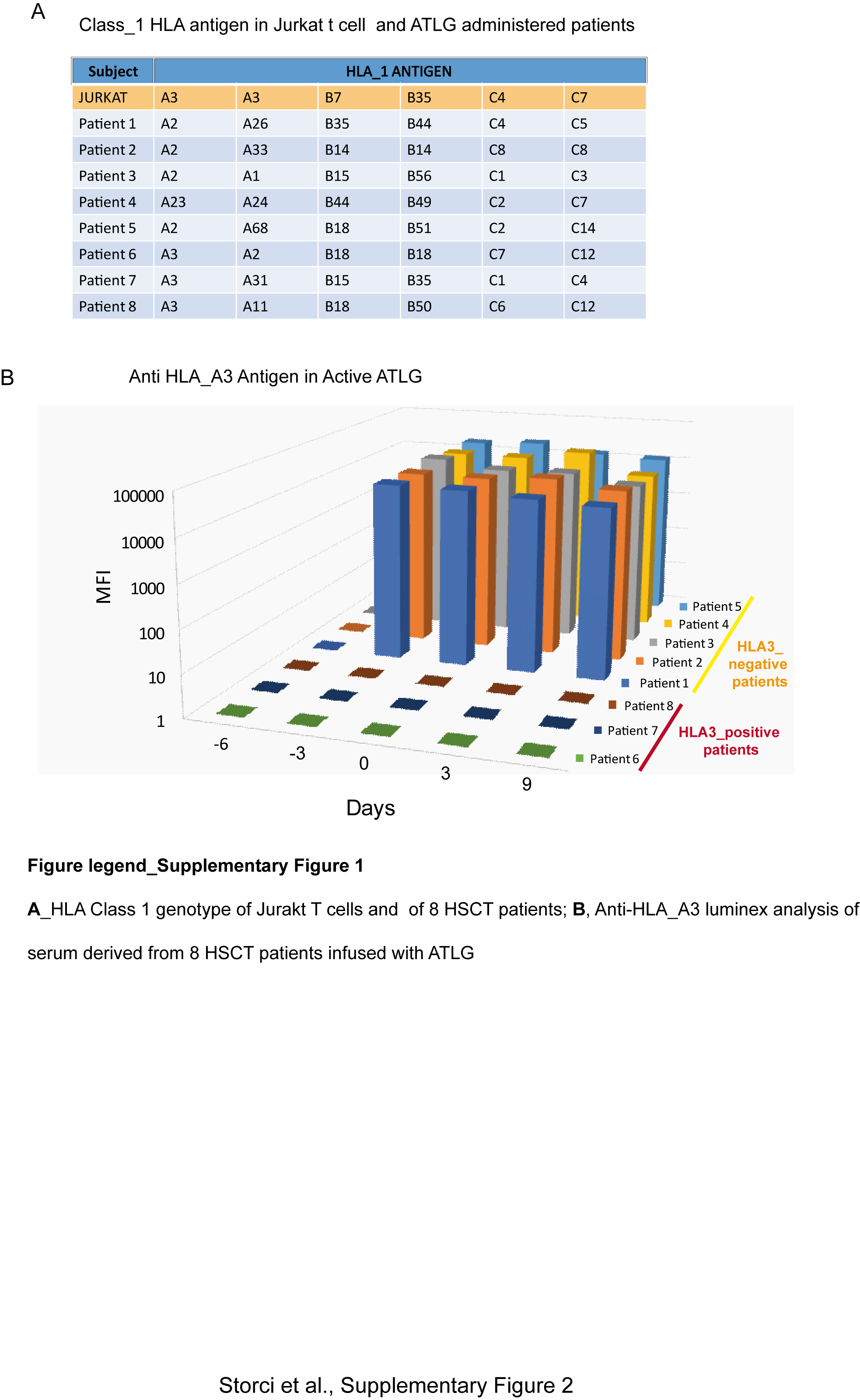

Supplement: Supplementary file 2 [file Image_2.tif]

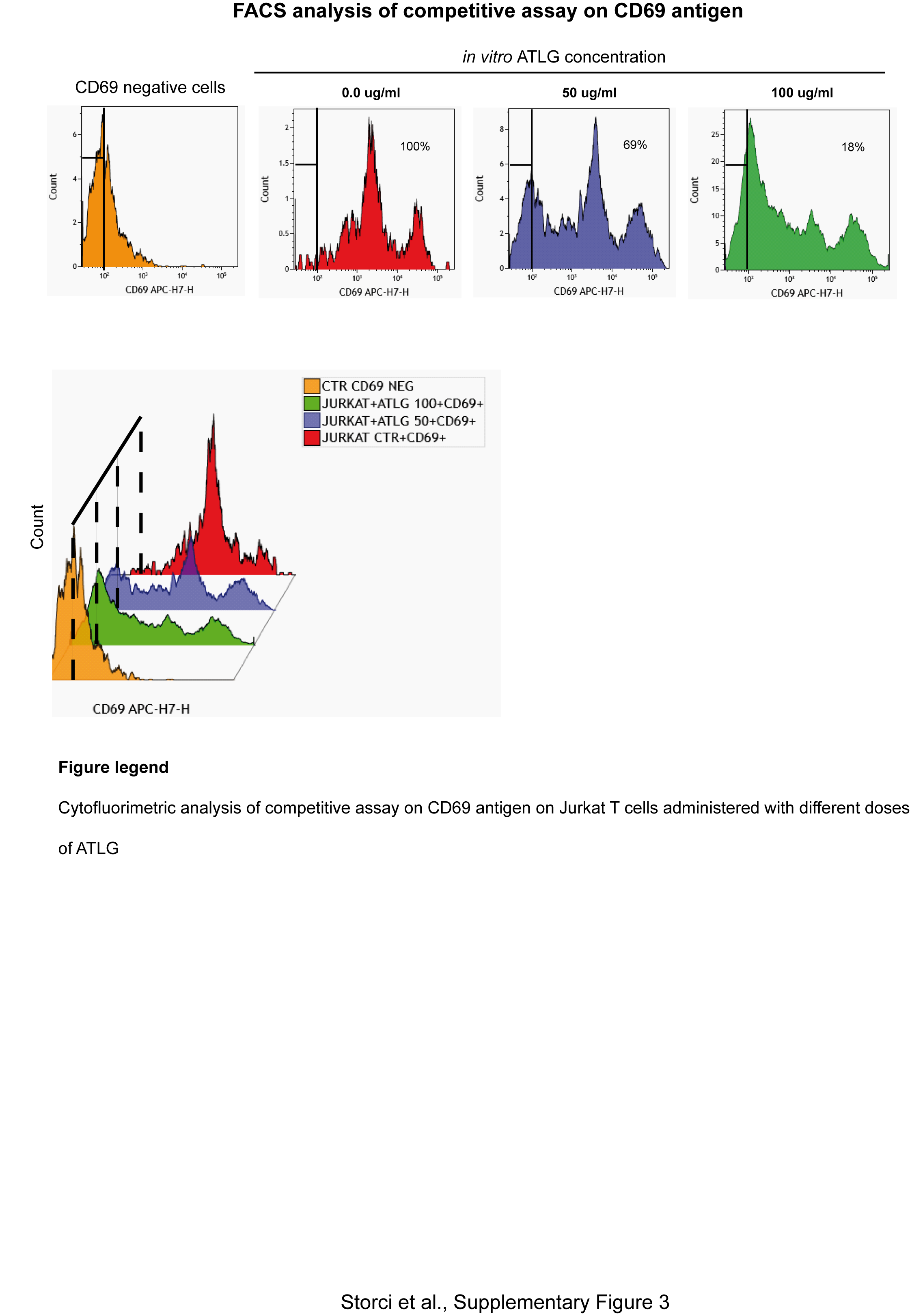

Supplement: Supplementary file 3 [file Image_3.tif]
